# The integrated National NeuroAIDS Tissue Consortium database: a rich platform for neuroHIV research

**DOI:** 10.1093/database/bay134

**Published:** 2019-01-08

**Authors:** Abigail J Heithoff, Steven A Totusek, Duc Le, Lucas Barwick, Gary Gensler, Donald R Franklin, Allison C Dye, Sanjit Pandey, Seth Sherman, Chittibabu Guda, Howard S Fox

**Affiliations:** 1 Department of Pharmacology and Experimental Neuroscience, College of Medicine, University of Nebraska Medical Center, Omaha, NE, USA; 2 Bioinformatics and Systems Biology Core, University of Nebraska Medical Center, Omaha, NE, USA; 3 Emmes Corporation, Rockville, MD, USA; 4 Department of Medicine, University of California, San Diego, La Jolla, CA, USA; 5 Department of Genetics, Cell Biology and Anatomy, University of Nebraska Medical Center, Omaha, NE, USA

## Abstract

Herein we present major updates to the National NeuroAIDS Tissue Consortium (NNTC) database. The NNTC's ongoing multisite clinical research study was established to facilitate access to ante-mortem and post-mortem data, tissues and biofluids for the neurohuman immunodeficiency virus (HIV) research community. Recently, the NNTC has expanded to include data from the central nervous system HIV Antiretroviral Therapy Effects Research (CHARTER) study. The data and biospecimens from CHARTER and NNTC cohorts are available to qualified researchers upon request. Data generated by requestors using NNTC biospecimens and tissues are returned to the NNTC upon the conclusion of requestors' work, and this external, experimental data are annotated and curated in the publically accessible NNTC database, thereby extending the utility of each case. A flexible and extensible database ontology allows the integration of disparate data sets, including external experimental data, clinical neuropsychological and neuromedical testing data, tissue pathology and neuroimaging data.

## Introduction

Advances in treatment and the development of combination antiretroviral therapy have reshaped the long-term outlook for individuals living with human immunodeficiency virus (HIV). Those infected with HIV are living longer, healthier lives than in the past and, thus, the disease profile is increasingly recognized as that of a chronic infection (1, 2). Research into the interaction of HIV disease with illnesses related to aging and chronic inflammation is critical to ensuring the health of the aging HIV-positive population. One long recognized complication related to HIV is the development of neurological disorders, involving the peripheral and central nervous systems (PNS and CNS) (3). These disorders include mild to moderate findings of neurocognitive disorders, HIV-associated dementia and peripheral neuropathy associated with HIV infection and neurotoxic antiretroviral treatment (ART) use (3, 4). Research into the chronic effects of HIV infection benefits from a longitudinal study design, which can be practically difficult to implement, and the complicated nature of the disease and clinical landscape requires a specialized set of assessments and batteries that is designed and administered by domain experts to explore the factors underlying patient outcomes. To address the particular needs of the neuroHIV research community, specialized clinical data and tissue repositories have been developed with the goal of providing relevant data and biospecimens for research (5).

The National NeuroAIDS Tissue Consortium (NNTC) is composed of four national clinical sites and a data coordination center (DCC) that work cooperatively as a resource for the neuroHIV research community. The NNTC provides HIV/AIDS investigators with clinically annotated data sets of ante-mortem information and biofluids as well as post-mortem tissues, with a focus on nervous system functional data and specimens (6). While the majority of subjects are HIV-infected, uninfected control subjects are also included. While the clinical sites have their own databases, the DCC maintains a central database and supports the functions of the NNTC by managing data and specimen requests from scientists. Recently, the DCC became responsible for housing the clinical research data from an additional study of HIV and the nervous system, the CNS HIV Antiretroviral Therapy Effects Research (CHARTER) study (comprised of six national clinical sites, three of which are also NNTC sites) (7) and external experimental data from CHARTER biospecimen requestors. The curation process implemented by the DCC identifies and leverages linkages between these disparate resources, adding value for research by providing access to an expanded clinical research population and previously generated experimental research data. This paper describes the approach taken by the NNTC–DCC for the creation of an integrated neuroHIV research database.

The clinical and external experimental research data sets provide researchers a rich platform for hypothesis generation, *post hoc* analyses and biospecimen request shaping. Integrating and harmonizing the CHARTER and NNTC clinical data increases the utility of the separate resources by providing synchronous access to both databases, together comprised of over 4000 unique participants studied at seven clinical sites across the United States. The studies use different enrollment criteria when identifying participants (6, 7), and integrating the clinical data sets allows researchers to interrogate a broader research population and avoid shortages of critical biospecimens (8). The increased population size and fully harmonized database structure also allow the NNTC–DCC to identify subcohorts of special research interest, including a subpopulation of virally suppressed individuals who underwent autopsy, a unique resource important for HIV cure research (9, 10). Integrating external experimental data from prior NNTC and CHARTER biospecimen requests also allows researchers to easily build on prior experimental findings and prevents redundancies across biospecimen requests (5).

## Description of clinical and experimental resources

The NNTC was established in 1998 to facilitate access to ante-mortem and post-mortem tissues and fluids for the international neuroHIV research community (6). The NNTC network of brain banks is charged with the collection of biofluids, tissues and data in a standardized fashion. As of September 2018, the study has enrolled 2840 participants with HIV and 338 participants without HIV. In addition to participants who donate tissues to the NNTC at death, many in the HIV+ cohort are enrolled into the longitudinal clinical study. For this group, cerebrospinal fluid and blood are collected longitudinally, brain and other vital organs are obtained and pathology is assessed post-mortem. This cohort also receives regular assessments to measure HIV disease progression, CNS and PNS symptoms, co-morbid and medical conditions and immunological and virological parameters. Demographic and selected HIV disease parameters are summarized in Table 1. By its nature as a brain bank, the NNTC largely enrolls participants with advanced HIV disease or those at a risk of death from other conditions (6).

**Table 1:** Selected demographic and HIV disease characteristics of the CHARTER and NNTC clinical research populations.

	CHARTER cross-sectional cohort		Active NNTC cohort		Longitudinal NNTC cohort		NNTC tissue bank cohort	
Demographics and HIV characteristics:	*N*	Percentage	*N*	Percentage	*N*	Percentage	*N*	Percentage
Gender								
Male	1222	76.5%	465	77%	1979	82%	754	81%
Female	375	23.5%	137	23%	445	18%	181	19%
Race/ethnicity^*^								
Black	771	48.3%	194	32%	789	33%	280	30%
White	634	39.7%	332	55%	1356	56%	556	59%
Hispanic (any race)	151	9.5%	177	30%	655	27%	253	27%
Other	41	2.6%	75	12%	272	11%	99	11%
Educational attainment								
Less than high school diploma/equivalency	293	18.5%	168	28%	680	32%	135	29%
High school diploma/equivalency	399	25.2%	114	19%	486	23%	126	27%
Some college/associate's degree	622	39.2%	183	31%	596	28%	134	28%
Bachelor degree	169	10.6%	80	13%	240	11%	38	8%
Graduate degree/graduate work	102	6.4%	54	9%	155	7%	40	8%
Current ARV medication use	1122	70.8%	556	92%	1940	80%	490	52%
HIV risk category								
IV drug use	167	14.1%	122	20%	598	25%	271	29%
Homosexual sexual contact	608	51.2%	285	47%	1109	46%	366	39%
Heterosexual sexual contact	358	30.2%	162	27%	538	22%	173	19%
Other	54	4.5%	33	5%	182	7%	125	13%
Demographics and HIV characteristics (cont.)	Value		Value		Value		Value	
Age								
25th percentile	38		42		38		41	
Median	43		51		44		48	
75th percentile	49		61		52		56	
CD4 count								
25th percentile	264		157		46		16	
Median	421		364		156.5		84	
75th percentile	463		594		371		247	
Log plasma viral load								
25th percentile	Undetectable		1.60		1.70		1.70	
Median	2.28		1.70		2.70		3.65	
75th percentile	4.06		2.79		4.53		5.12	

The CHARTER cross-sectional study includes all CHARTER participants (those who did and did not recruit into a longitudinal substudy). The NNTC active cohort is currently enrolled and being followed on study. The longitudinal NNTC cohort includes both members of the active cohort and inactive participants. The NNTC tissue bank cohort includes all study participants who are deceased with autopsy. ^*^The race/ethnicity data for the CHARTER study are collected in a single, mutually exclusive question; the data for NNTC are collected using separate race and ethnicity questions and are not mutually exclusive.

**Figure 1:**
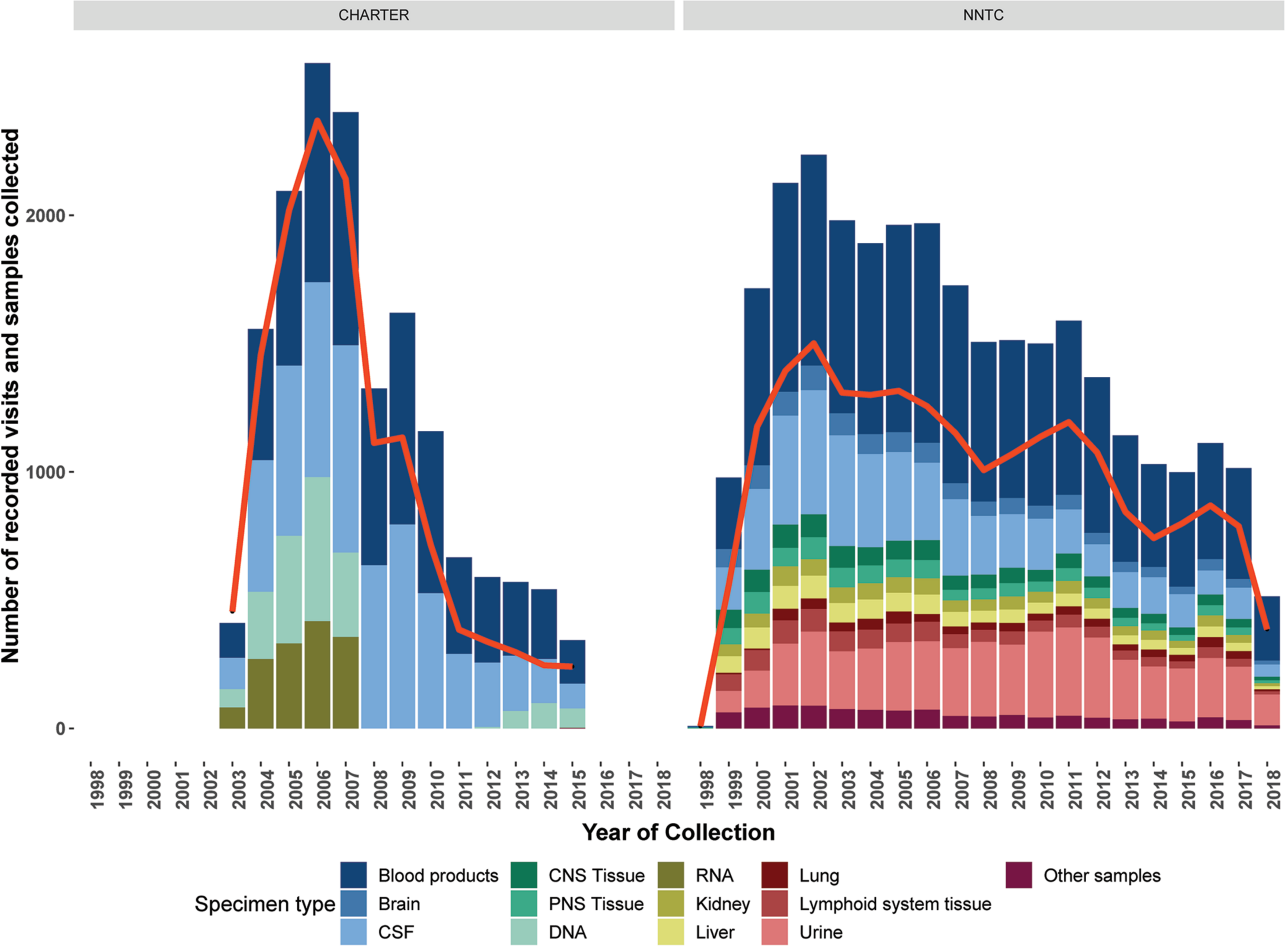
Number of data collection visits conducted (red line) and number and types of biospecimens collected of type (colored bars) for each year of the CHARTER and NNTC clinical research studies. Data and biospecimen collection for the NNTC clinical research study is ongoing, data are through September, 2018.

The CHARTER study used minimal exclusion criteria during recruitment and screening and thus represents a broader range of participants than the NNTC cohort (7). A total of 1608 HIV+ participants enrolled into the CHARTER study from 2003–07. At their first visit, participants received a core battery of assessments, many of which overlap with the NNTC neuropsychological and neuromedical assessments (7). ~45% of CHARTER participants were recruited into at least one of six longitudinal substudies after their first visit. These substudies utilized the core battery of assessments, plus study specific tests. The study specific tests within the longitudinal substudies were tailored to examine fluctuating patterns of complications in the context of ART, effect of metabolic disorders induced by ART on neurological conditions, HIV history and development of peripheral neuropathy, HIV penetration into the CNS in the early stages of infection, effect of viral genetics on neurocognitive dysfunction and utility of neuroimaging techniques to capture physiological changes induced by HIV infection. In addition to clinical data, CHARTER collected blood, urine, cerebrospinal fluid, intraepidermal nerve fiber density (for the peripheral neuropathy substudy) and structural MRI data (for the neuroimaging substudy) (7). The number of data collection visits and types of specimens collected over time for CHARTER and the NNTC studies is shown in Figure 1. Both CHARTER and the NNTC are focused on studying the presentation of neurocognitive disease in the context of HIV and therefore the research domains within each study extensively overlap.

Biospecimens and data from the NNTC and CHARTER cohorts are available to the neuroHIV research community qualified researchers by request and represent a valuable research repository. The time span from request submission to approval is 2–8 weeks. The DCC and requestor work cooperatively to shape specimen and data requests to best leverage the resource and to address the requestor's research needs. The shaping process may involve the advice of domain experts, the NNTC Allocations Committee and the Steering Committee. Requestors also sign a data-sharing agreement, acknowledging their responsibility to submit published, experimental research data to the DCC. The DCC tracks the research progress of each requestor through routine follow-up communications and, upon publication, helps to facilitate the requestor's submission of experimental data to the central repository.

**Figure 2:**
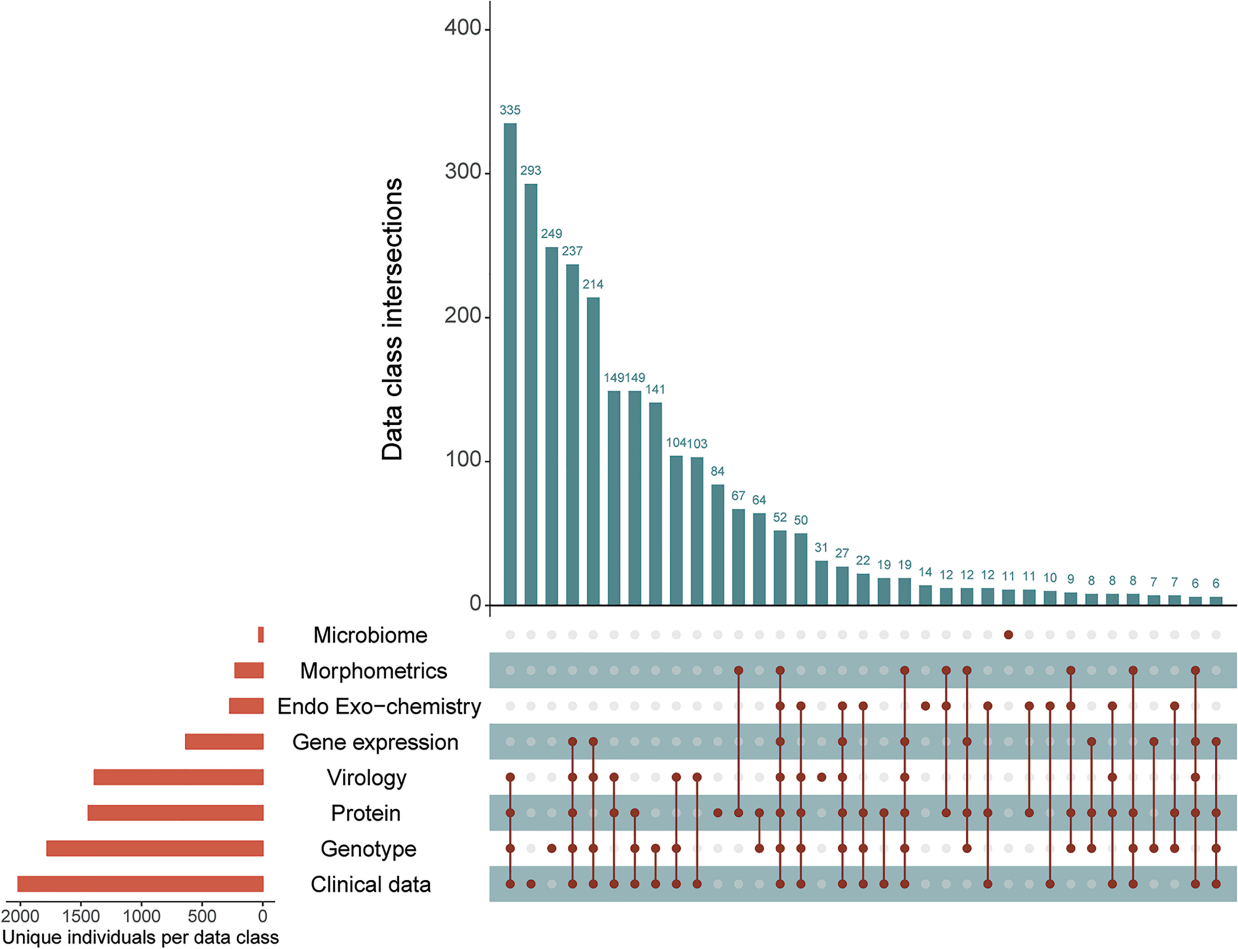
Participant intersections in the external, experimental database measurement classes. Orange bars show the number of unique individuals represented in that measurement class in the external database. Vertical, blue bars show the number of unique participants co-represented across the measurement classes (connected dots) in the experimental database.

Submitted data are annotated according to the experimental database ontology, which characterizes data by measurement type, assay method, specific technology utilized, tissue of interest and assay target (5). The experimental data received in September 2018 include 686 unique data types organized along the five data axes for 2573 unique NNTC and/or CHARTER participants. Integration of the clinical NNTC and CHARTER databases with external experimental data from biospecimen requestors leverages natural linkages inherent to common resource elements. The applied ontology allows researchers to identify previously unrecognized linkages between different external, experimental studies. Figure 2 shows the number of unique participants with data spanning multiple measurement classes in the experimental database.

## Requirements for infrastructure and core metadata elements

The focus of integrating the NNTC database resources was on ensuring the security while improving the accessibility and discoverability of data through the NNTC–DCC's online portals. To ensure data security, the online portal for the NNTC central database is deployed in a secure application service provider (ASP) environment and communications are encrypted. For external security, the servers storing the clinical research data are behind multiple port-based and application-based firewalls. This architecture ensures that only authorized users have access to data and applications. New users are given access to project areas as work and skills that is dictate through access-based enumeration. The CHARTER and external experimental data query tool is deployed in a secure ASP environment with Secure Sockets Layer (SSL) encryption. To ensure data security, a dynamically updated flat file is used so end users may access and download only the data displayed on the query tool grid view. To ensure anonymization of participant data, query tool data fields have been converted to range values, top-coded or otherwise obfuscated.

**Figure 3:**
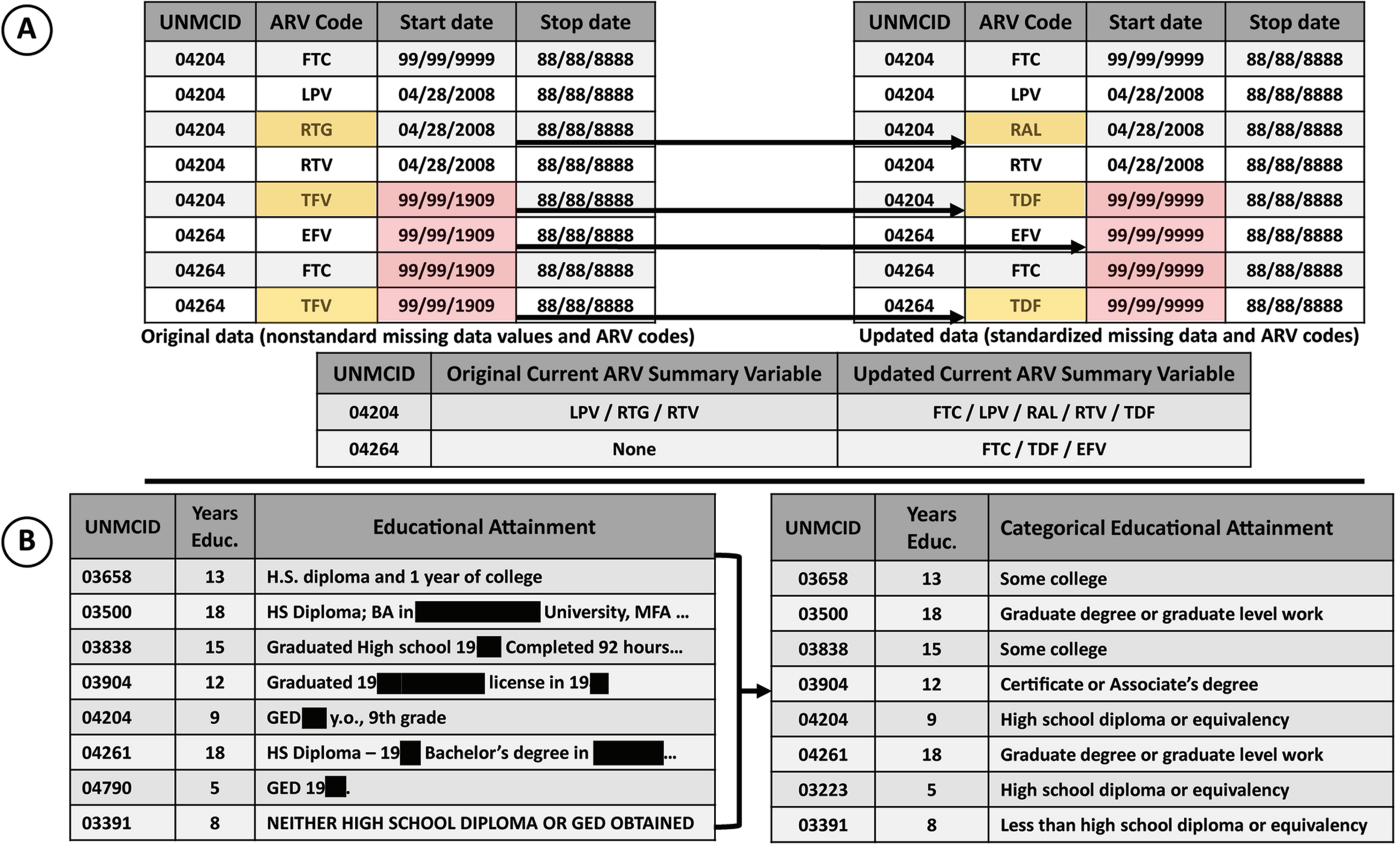
**A**) The effect of standardized missing data and ARV codes on downstream ‘current ARV regimen’ variable. ARV codes were standardized to the NIH ARV drug name abbreviations (e.g. for the participants with IDs 04204 and 04264 TFV was used as an abbreviation for tenofovir disoproxil fumarate, which was then standardized to TDF). Nonstandard missing start dates for ARVs resulted in an underestimation of drugs in the regimen. Correction of the missing data to the correct code (99/99/9999) shows that three ARVs were included in the drug regimen of participant with ID 04264, where previously none were included. **B**) Educational attainment was collected using an open-ended question item. The raw data contained personal information (concealed here) and were not readily useful for analytical models. Natural language processing was used to operationalize an ordered categorical educational attainment variable (right) from the raw data (left).

The DCC centralized database for the NNTC study was begun in 2003, and the CHARTER integration with the NNTC began in 2015. As part of this integration, whenever possible, the sophisticated, centralized CHARTER database was harmonized with the NNTC clinical database by processing the data using the same algorithmic logic checks applied to NNTC data to detect and correct out-of-range and inconsistent data. Imposing the NNTC database architecture on the extant CHARTER data added a value to the database. For example, the NNTC database uses a standard set of antiretroviral (ARV) codes recommended by the National Institutes of Health (NIH). The CHARTER data used multiple drug codes to represent the same active ARV drug, depending on era and formulation. Standardization reduces the complexity of the data and allows a more straightforward query structure. Missing data were also standardized to a single code, to simplify the algorithmic processing needed to generate summary variables (Figure 3A). Additionally in CHARTER, educational attainment was collected using free-text, verbatim responses and a continuous variable representing years of education. In the NNTC database, education was collected as a nominal variable representing relative educational attainment and a continuous variable of years of education. To harmonize these database elements, the DCC used natural language processing (NLP) and double-blind validation to codify an educational attainment variable from the free-text data collected in CHARTER (Figure 3B). Harmonizing the education variables across the two clinical data sets greatly simplifies analysis of these data using statistical models. Additionally, the NLP scripts used to convert the free-text data to a categorical variable obfuscate a high level of detail about the participant's history, while providing additional information about relative educational attainment to contextualize years of formal education. For example, neither of the participants identified as 04790 and 03391 completed 12 years of formal education; however, the participant identified as 04790 (who finished fewer years of formal education) did complete his General Educational Development (GED), while the participant identified as 03391 did not (Figure 3B).

To improve the accessibility of the clinical and experimental research data sets, both an NNTC query tool and a separate CHARTER query tool have been created for data exploration (located at www.nntc.org and https://neuroaids-dcc.unmc.edu). These query tools allow researchers to view the clinical and experimental data available from the NNTC or CHARTER cohorts and download associated data sets in a standardized format. Because CHARTER and NNTC protocols are not always comparable, some of the data values extant in one database may not exist in the other or data values may be collected in non-standardized ways. The query tools for the two cohorts were selected with this in mind and thus leverage synchronous data elements within the clinical databases to ensure parallel query tool variables. The use of these selected data across the query tools ensures that a query results from the two tools can be easily and meaningfully combined. To further integrate the databases, a unified participant identification variable was developed. This allows external, experimental data to be quickly merged with the NNTC and CHARTER clinical research data. The development of a central identification variable has the added benefit of stripping the geolocation information inherent due to format differences between the seven clinical research sites. Experimental data submitted by users of the NNTC resource are curated and annotated by the DCC. The curated data are available for download, and each archive includes the experimental study data, a data dictionary, a study summary and methods write-up. After a data set is downloaded, the researcher who has downloaded it is connected via email message to the original data submitter to facilitate collaboration and ensure the data is properly analyzed.

The utility of the external experimental database is greatly extended by integration with external databases. The external experimental data is integrated with the HUGO Gene Nomenclature Committee database (11) and with the Gene Expression Omnibus (GEO) repository for the experimental data gene search tool. This tool allows researchers to query GEO profiles associated with NNTC and CHARTER studies for specific genes of interest. The experimental database publication search tool is also integrated with GEO. The GEO PubMed ID is also used to identify publications within the external experimental database, and the publication search tool is integrated with PubMed such that publications can easily be identified using the query tool.

## Conclusion and future directions

Near-future development on the NNTC and CHARTER public data access portal will include the development of an online web application for data visualization. This tool will allow researchers to plot data into customized graphics in their browser, without having to download or manually manipulate the data. These tools are intended to provide a user-friendly platform for data exploration. The visualization tools are developed in Shiny, an open-source R package for building interactive web applications (https://shiny.rstudio.com). Controls for the tool will be predefined in-house, whereas output options will be defined by the user. As with the query tool, data will be sourced from data files housed separately from the parent database in an encrypted virtual space.

Other steps are being taken to simplify data exploration of the NNTC and CHARTER cohorts. Ultimately, the NNTC and CHARTER query tools, currently available as separate resources, will be federated. This will allow researchers to easily browse all the data and biospecimens available from both cohorts without having to download and manually combine results. This federation will rely on the development of parallel query tool variables and unified metadata resources (data dictionaries, codebooks and protocols)—this work is ongoing at the DCC.

## Notes

**Database URL**: https://neuroaids-dcc.unmc.edu
